# The coming and going of Batesian mimicry in a Holarctic butterfly clade

**DOI:** 10.1186/1741-7007-8-122

**Published:** 2010-09-15

**Authors:** Konrad Fiedler

**Affiliations:** 1 Department of Animal Biodiversity, University of Vienna, Rennweg 14, 1030 Vienna, Austria

## Abstract

A study using phylogenetic hypothesis testing, published in *BMC Evolutionary Biology*, suggests that non-mimetic forms of the North American white admiral butterfly evolved from a mimetic ancestor. This case might provide one of the first examples in which mimicry was gained and then lost again, emphasizing the evolutionary lability of Batesian mimicry.

See research article http://www.biomedcentral.com/1471-2148/10/239

## 

Comparative studies of butterfly wing patterns by naturalists such as Henry Bates, Roland Trimen and Fritz Müller marked the birth of the scientific concept of mimicry right at the onset of the Darwinian age. Even today, the factors governing the origin, maintenance and dynamics of mimicry systems remain a challenge for evolutionary biologists, and butterfly wing patterns continue to provide prime models for developing and testing new ideas concerning the mechanisms governing how mimetic phenotypes arise and what factors may regulate their maintenance [1]. The emergence and maintenance of Batesian mimicry, in which palatable mimics share conspicuous warning color patterns with unpalatable models that are protected from predation by their aposematic pattern, is particularly intriguing in evolutionary terms. Here, fitness benefits will accrue to the non-toxic mimic only as long as the toxic model remains present, and in large enough numbers, to ensure that predators are familiar with it and are thus warned off by its characteristic appearance. Otherwise, selection should favor the disappearance of palatable mimics, which suffer from higher predation risk than inconspicuous phenotypes [2].

White admirals, that is, the Holarctic butterfly genus *Limenitis *(Nymphalidae), have been the target of research into the function and evolution of mimicry for more than 40 years. The genus comprises about 25 species in Asia, Europe and North America. Most of them show disruptive wing coloration [3]: dark brown with white bands stretching across fore and hind wings, and undersides similar. Within the four North American species, sometimes referred to as subgenus *Basilarchia*, two radically different phenotypes occur that exemplify two different mimicry syndromes. On the one hand, *Limenitis archippus*, the viceroy, is orange colored and forms a Müllerian mimicry ring with toxic *Danaus plexippus *(the monarch) and *D. gilippus *[4]. In a Müllerian mimicry ring, all species share a common warning color pattern, and since they are all unpalatable to predators, they collectively benefit from this common signaling. On the other hand, *L. arthemis *(the white admiral) comprises an experimentally proven example of Batesian mimicry [5]. Its northern two subspecies, *arthemis *and *rubrofasciata*, show the disruptive coloration usual for the genus and are non-mimetic. However, the southwestern (*arizonensis*) and southeastern (*astyanax*) subspecies are bluish without white bands, and with conspicuous red dots ventrally. They are mimics of the toxic pipevine swallowtail *Battus philenor*. The four forms of *Limenitis arthemis *freely interbreed in nature as well as in captivity and thus belong to the same species under the biological species concept.

The past 5 years have seen an interesting controversy as to whether mimetic forms in the *L. arthemis *complex have evolved once (monophyletic mimicry hypothesis (MMH); Figure 1b), or whether the non-mimetic *arthemis *phenotype might constitute an example of the reversion to an ancestral phenotype from a mimetic one (reversion hypothesis (RH); Figure 1a). A first sequence-based phylogenetic analysis [6] did not support the MMH, but soon after, Savage and Mullen [7] concluded the MMH to be more appropriate on the grounds of amplified fragment length polymorphism (AFLP) data, disputing support for the RH obtained from mitochondrial sequence data [8]. This controversy might appear of little general significance, were it not for the fact that evidence for a reversion from a mimetic to an ancestral phenotype is extremely rare so far [2].

**Figure 1:**
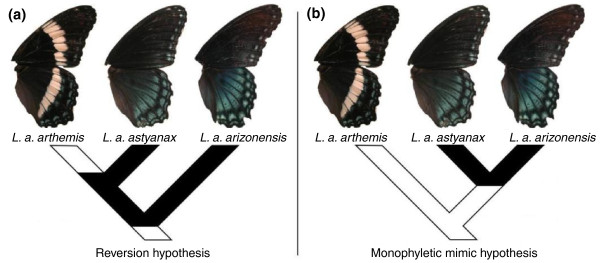
**Two contrasting hypotheses of mimicry evolution in the *Limenitis arthemis *species complex**. **(a) **According to the reversion hypothesis, mimetic *L. a. astyanax *is sister to non-mimetic *L. a. arthemis*. Under this hypothesis, the mimetic phenotype arose in the common ancestor to all *L. arthemis *and was subsequently lost in the *L. a. arthemis *lineage. **(b) **In contrast, the monophyletic mimic hypothesis predicts that the mimetic lineages *L. a. astyanax *and *L. a. arizonensis *are most closely related to each other and Batesian mimicry evolved only in the stem group of these two subspecies. Recent phylogenetic hypothesis testing [9] provided evidence in favor of the reversion hypothesis. Figure modified from [9].

## Phylogenetic hypothesis testing allows for new insights

In a study recently published in *BMC Evolutionary Biology*, Oliver and Prudic [9] now revisit the case. They use sequence information from eight nuclear loci combined with coalescent simulation of gene trees to evaluate a range of models of population structure and evolutionary history. By using multiple loci they compensate, at least partially, for the problem of gene-tree/species-tree discrepancies. The main difference from earlier approaches, however, is that Oliver and Prudic use sophisticated statistical models to measure how well their simulations fit the empirical data. Parameters for these 15 models were estimated divergence times (for species evolution) and migration rates (for population structure), taken from earlier studies of the same species. The advantage of this approach is that explicit models for contrasting evolutionary scenarios are compared with each other. Hence, inference is based on rigorous statistical tests of explicitly formulated alternatives. Oliver and Prudic found that the MMH had to be rejected: the only model that fitted the data in all aspects was a scenario that assumes moderate migration rate of the butterflies plus divergence times of about 655,000 years for the split of *arthemis *from *astyanax*, and 1,075,000 years for the split of the western *arizonensis *from the eastern (*arthemis *+ *astyanax*) clade.

Is this the end of the story? Certainly not. First, even if monophyly of the two mimetic forms now seems to be rejected with good support, this is not yet firm evidence for a reversion of the ancestral phenotype from the mimetic one. Two independent gains of Batesian mimicry in *astyanax *and *arizonensis *could still have occurred, while *arthemis *just retained the plesiomorphic character state. This scenario would require two steps in character evolution - exactly the same number as one gain of mimicry at the base of the *arthemis *complex, and one loss subsequently at the split between *arthemis *and *astyanax*. To decide conclusively between these competing scenarios a better understanding of the genetic basis and physiological processes that determine the two different phenotypes in the *L. arthemis *group will be required. While *prima facie *butterfly wing patterns might be seen as complex characters, with a low likelihood of convergent evolution, in fact most cases of butterfly mimicry are based on increased melanism. Major 'macro-evolutionary' changes in wing color patterns could thus be controlled by very few genes, or even one single major developmental gene [10,11]. In that case, convergent evolution of similar melanic (and at the same time mimetic) phenotypes remains a plausible alternative. One obvious approach to a better understanding is therefore to unravel the developmental pathways that lead to mimetic phenotypes and their genetic basis, by genomic analysis, for example [11].

Second, the phylogenetic and statistical analyses of Oliver and Prudic are not immune to criticism. The sample sizes for some genes were very small. To obtain a more comprehensive picture of the history of *Limenitis *phenotypes in North America, including possibly complex patterns of gene flow, a thorough phylogeographic study would be required, using a larger number of populations from the entire range of the complex, a larger number of genetic markers, and incorporating the allied species *L. weidemeyerii *and *lorquini *with a larger number of samples. That last requirement seems important, as hybrids between *arthemis *and these two relatives do occur. If introgression between species and subspecies has been a significant phenomenon in the phylogeographic history of North American white admirals, it will have left traces in the genetic architecture as well as in the phenotypes of *L. arthemis *- which would be likely to go unnoticed in too small samples.

This new study on the evolution of mimicry in butterflies exemplifies the fact that, even in putatively well-studied cases, many questions about evolutionary processes remain to be settled. The application of phylogenetic hypothesis testing allows a great step forward as it provides measures of support that can be compared across competing scenarios. As with all statistical models, the problem of parameterization remains. Even for parameters such as divergence times and migration rates, empirical estimates are often unrealistic - for example, in the absence of fossil evidence for calibration or when information on a species' population biology is scant. Nevertheless, with increasing availability of computing facilities and pertinent software the lesson to be learned is that explicit comparison across competing phylogenetic hypotheses is now one, among many, approaches to unraveling the evolution and function of the fascinating diversity of butterfly wing patterns.

## References

[B1] Mallet J Joron M Evolution of diversity in warning color and mimicry: polymorphisms, shifting balance, and speciation Annu Rev Ecol Syst 1999 30 201 233 10.1146/annurev.ecolsys.30.1.201

[B2] Harper GR Pfennig DW Selection overrides gene flow to break down maladaptive mimicry Nature 2008 451 1103 1106 10.1038/nature06532 18305543

[B3] Ruxton GD Sherratt TN Speed MP Avoiding Attack: The Evolutionary Ecology of Crypsis, Warning Signals and Mimicry 2004 Oxford, UK: Oxford University Press

[B4] Ritland DB Comparative unpalatability of mimetic viceroy butterflies (*Limenitis archippus*) from four south-eastern United States populations Oecologia 1995 103 327 336 10.1007/BF00328621 28306826

[B5] Platt AP Coppinger RP Brower LP Demonstration of the selective advantage of mimetic *Limenitis *butterflies presented to caged avian predators Evolution 1971 25 692 701 10.2307/2406950 28564787

[B6] Mullen SP Wing pattern evolution and the origins of mimicry among North American admiral butterflies (Nymphalidae Limenitis) Mol Phylogenet Evol 2006 39 747 758 10.1016/j.ympev.2006.01.021 16500119

[B7] Savage WK Mullen SP A single origin of Batesian mimicry among hybridizing populations of admiral butterflies (*Limenitis arthemis*) rejects an evolutionary reversion to the ancestral phenotype Proc Biol Sci 2009 276 2557 2565 10.1098/rspb.2009.0256 19369265 PMC2686656

[B8] Prudic KL Oliver JC Once a Batesian mimic, not always a Batesian mimic: mimic reverts back to ancestral phenotype when model is absent Proc Biol Sci 2008 275 1125 1132 10.1098/rspb.2007.1766 18285285 PMC2602694

[B9] Oliver JC Prudic KL Are mimics monophyletic? The necessity of phylogenetic hypothesis tests in character evolution BMC Evol Biol 2010 10 239 10.1186/1471-2148-10-239 20682073 PMC3020633

[B10] ffrench-Constant R Koch PB Boggs CL, Watt WB, Ehrlich PR Mimicry and melanism in swallowtail butterflies: toward a molecular understanding Butterflies: Ecology And Evolution Taking Flight 2003 Chicago: Chicago University Press 259 279

[B11] Ferguson L Lee SF Chamberlain N Nadeau N Joron M Baxter S Wilkinson P Papanicolaou A Kee T-J Clark R Davidson C Glithero R Beasley H Vogel H ffrench-Constant R Jiggins C Characterization of a hotspot for mimicry: assembly of a butterfly wing transcriptome to genomic sequence at the HmYb/Sb locus Mol Ecol 2010 19 Suppl 1 240 254 10.1111/j.1365-294X.2009.04475.x 20331783

